# Mosses on Geopolymers: Preliminary Durability Study and Chemical Characterization of Metakaolin-Based Geopolymers Filled with Wood Ash

**DOI:** 10.3390/polym15071639

**Published:** 2023-03-25

**Authors:** Michelina Catauro, Veronica Viola, Alberto D’Amore

**Affiliations:** Department of Engineering, University of Campania “Luigi Vanvitelli”, Via Roma n. 29, 81031 Aversa, Italy; michelina.catauro@unicampania.it (M.C.); alberto.damore@unicampania.it (A.D.)

**Keywords:** geopolymers, wood ashes, mechanical properties, chemical properties, green cements

## Abstract

Burning wood is estimated to produce about 6–10% of ash. Despite the possibility of recycling wood ash (WA), approximately 70% of the wood ash generated is landfilled, causing costs as well as environmental pollution. This study aims to recycle WA in an alternative way by inserting it as filler in geopolymeric materials. Here, metakaolin, NaOH, sodium silicate, and WA are used to realize geopolymers. Geopolymers without and with 10, 20 and 30% of WA are synthesized and characterized after 7, 14, 28 and 56 days. The article’s study methods are related to geopolymers’ chemical, biological and mechanical properties. The geopolymers synthesized are compact and solid. The pH and conductivity tests and the integrity and weight loss tests have demonstrated the stability of materials. The FT-IR study and boiling water test have confirmed the successful geopolymerization in all samples. The antibacterial analysis, the moss growing test and the compressive strength test have given a first idea about the durability of the materials synthesized. Furthermore, the compressive strength test result has allowed the comparison from the literature of the specimens obtained with the Portland cement (PC). The results obtained bode well for the future of this material.

## 1. Introduction

Wood ash (WA) is the organic residue that remains after the combustion of wood and wood products [1]. Usually, when plant materials are subjected to high-temperature treatment, this burns away almost all the organic parts, leaving only the inorganic component as mineral salts. According to the estimate, burning wood produces about 6–10% of ash [2]. However, wood ash’s physical and chemical properties determine its beneficial uses, depending on the wood’s species and combustion methods [3]. Some authors reported that the chemical composition of ash varies also among the growing conditions (soil type and climate), the age (decayed vs. non-decayed trees), and the types of bark in tree species (decorticating vs. fibrous, stringy barks) [4,5]. When wood is burned, the ash produced can be separated into two main types: bottom and fly ash [6]. The former is obtained from the base of the combustion chamber. The latter is subdivided into coarse and fine fractions and refers to the portion of the ash that escapes up the chimney or stacks in it [7]. There are several studies in which both fly and bottom ash are used and recycled [8,9,10,11]. However, despite the possibility of recycling wood ash, approximately 70% of the wood ash generated is landfilled; 20% is applied on land as a soil supplement, and only the remaining 10% has been used for miscellaneous applications including construction materials, metal recovery and pollution control [2].

Nowadays, landfilling costs are increasing due to strict environmental regulations and the limited availability of landfill space. Furthermore, some heavy metals and/or high alkalinity in wood ash may limit its application on land under stricter environmental regulations. In light of these, it is essential to develop beneficial uses for wood ash to meet the challenges associated with its disposal [12].

In Italy, as in other European countries, the estimation of wood consumption and ash production is unclear [13]. However, several surveys suggested that the Italian amount of wood consumption is much higher than that indicated by official sources [14]. Despite that, it is commonly recognized that wood biomasses play a relevant role in Italy. Moreover, the Italian dependence on the international biomass market has significantly increased over time [15,16].

Another source of wood ash in Italy is represented in pizza making. Italy is one of the most famous countries for making Neapolitan pizza, a renowned Italian food recognized as one of the traditional specialties guaranteed (TSG) by European Commission Regulation no. 97/2010, which should be exclusively baked in wood-fired ovens [17]. Thus, the production activities of artisanal pizza in restaurants, pizzerias, bars, delicatessens and takeaway restaurants result in the widespread use of wood-fired ovens, and consequently, a contingent wood ash production. Therefore, recycling wood ash could be a way to avoid waste and support the local circular economy.

While supporting the circular economy leads to economic benefits, it is also a huge step toward protecting the environment. Today’s policies are very much focused on environmental protection, and in the field of construction, efforts are being made to minimize CO_2_ emissions that are the main cause of global warming. Some examples of environmentally sustainable materials from this point of view are represented by low-carbon cement, sulphoaluminate cement, etc. [18,19]. Among all these new materials, geopolymers, as substitution for cement, are also attracting attention. Geopolymers are not only eco-sustainable materials due to their low consolidation temperature and consequently their low carbon emissions, but also because secondary raw materials can be used for their production [20].

To date, various wastes have been incorporated with more or less success within the geopolymeric matrix, e.g., waste glass, volcanic fly ash, blast furnace slag [21,22,23,24].

This research focuses on the possibility of including wood ash as a filler in the geopolymers. The main goal of this study is to synthesize a material, using waste, that can be used as a substitution for cement in the external reinvestment of buildings and architecture. To obtain the desired result, geopolymers without and with 10, 20 and 30% of wood ash were synthesized and characterized after 7, 14, 28 and 56 days. Different parameters and tests were realized to study their chemical, biological and mechanical characteristics. Integrity test, weight loss test, pH and conductivity measurements were made to understand the stability of the materials. In addition, Fourier-Transform Infrared Spectroscopy (FT-IR) analysis and water boiling test were conducted to evaluate the geopolymerization process; antibacterial activity, moss growing test and mechanical compressive strength were realized to obtain information about their mechanical and biological proprieties and to obtain information about the possible durability of the materials. All the analyses performed had the aim of verifying the theoretical feasibility of using this material in the designed application.

## 2. Raw Materials and Test Methods

### 2.1. Raw Materials

The precursor aluminosilicate powder used for the synthesis was the metakaolin (MK) purchased from IMCD Deutschland GmbH & Co. (Köln, Germany); while the filler, the wood ash (WA), was collected from a local restaurant in Aversa, Italy. The activation of the precursor was prepared with a mixture of sodium silicate (SiO_2_/Na_2_O = 3.3) and NaOH in pellets. The Prochin S.r.l Company (Caserta, Italy) supplied the former; the latter was supplied by Sigma Aldrich (Milan, Italy). Acetone (C_3_H_6_O), Potassium bromide (KBr), both of analytical grade, and MilliQ water were used for the sample analyses. All the reagents were purchased from Sigma Aldrich (Milan, Italy).

### 2.2. Chemical Composition of Raw Materials

#### 2.2.1. ED-XRF

The chemical components of the metakaolin powder and the wood ash were determined by Energy Dispersive X-Ray Fluorescence (ED-XRF) spectroscopy with a Shimadzu Spectrometer EDX-720 (GmbH, Duisburg, Germany). The machine was equipped with 50 W Rh target X-ray tube, a high-energy resolution Si (Li) detector and five primary X-ray filters. The detections were performed in the ranges of Na-Sc and Ti-U.

#### 2.2.2. TOC

Total Organic Carbon (TOC) and Inorganic Carbon (IC) on WA were determined according to UNI EN 15936:2012-Method A [25] to obtain information on the amount of unburned organic matter. Sample powders were analyzed by using a Total Organic Carbon Analyzer (TOC-Vcsh) coupled with a Solid Sample Module (SSM-5000A) produced by Shimadzu (Milan, Italy). The obtained results were expressed as TOC and IC percentages.

### 2.3. Experimental Process

Geopolymers specimens were prepared both without (GP) and with different percentages of wood ash (GP10%WA, GP20%WA, GP30%WA). The GP composition was realized starting from [26], with the following ratios: SiO_2_/Al_2_O_3_ = 4, Na_2_O/Al_2_O_3_ = 1 and H_2_O/Al_2_O_3_ = 13. The sample compositions and the water/solid ratio (W/S) are listed in Table 1.

**Figure 1:**
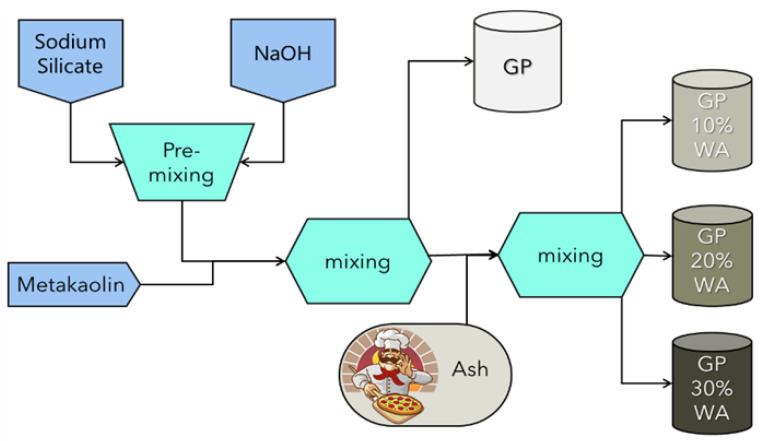
Flowchart showing the synthesis process of all the samples.

A graphical representation of the synthesis process is shown in the flowchart below (Figure 1). The first step for each synthesis was the dissolution of the NaOH pellets into the sodium silicate solution using a plastic beaker on a magnetic stirrer. After the complete dissolution of NaOH pellets and its cooling, the solution was gently added into the planetary mixer (Aucma 1400W, Acuma Co., Ltd., Qingdao, Shandong, China) previously loaded with the metakaolin. The GP sample was stirred for 15 min at a moderate speed before pouring the mixture into the different molds. After the filling procedure, all the molds were tapped on the work surface to eliminate as many air bubbles as possible and were covered with plastic film to avoid water evaporation. Next, the storage was done in the oven simulating the room temperature (25 °C) for 24 h. GP10%WA, GP20%WA and GP30%WA synthesis were conducted similarly: the mixer was loaded with metakaolin and the alkaline solution. After 10 min of stirring with a moderate speed, the wood ashes, previously sieved (65 < x < 79 μm), were added to the system and the stirring continued for another 5 min. The next steps were the same as for the GP samples.

### 2.4. Physicochemical Stability

The physicochemical stability of the geopolymer samples was verified with indirect measurements proposed in the literature [27,28]. The ionic conductivity, pH, integrity and weight loss tests were carried out according to procedures listed in the Supplementary Materials. Furthermore, the density of the synthesized materials was calculated using MQ water, a Class A cylinder and applying the Formula (1) where δ is the density, *m* is the mass of the sample and *V_f_* and *V_i_* are the final and initial volume of the water.

(1) $$\;\delta =\frac{m}{V_{f}-V_{i}}$$

### 2.5. FT-IR Analysis

FT-IR analysis was performed with the Prestige21 Shimadzu machine equipped with a DTGS KBr (deuterated triglycine sulfate with potassium bromide windows) detector. The resolution was 2 cm^−1^ (60 scans) with a frequency range between 400 and 4000 cm^−1^. Before the analysis, KBr disks were realized using 3.00 mg of the sieved sample and 197.00 mg of KBr. FT-IR spectra were elaborated by IR-Solution (v.160, Shimadzu, Milan, Italy) and Origin software (v.2022b, OriginLab Corporation, Northampton, MA, USA).

### 2.6. Antibacterial Activity

The synthesized samples’ antibacterial activity was evaluated using the Kirby–Bauer test. This test was performed by choosing a gram-positive and a gram-negative bacterium, *Staphylococcus aureus* (ATCC 25923) and *Escherichia coli* (ATCC 25922), respectively. The procedure involved the preparation of the samples and culture media, activating bacterial strains, and subsequently, their plating and incubation. Finally, the measurements of the inhibition halos’ diameters (IHDs) occurred. The culture media, TBX Medium (Tryptone Bile X-Gluc) (Liofilchem, Roseto degli Abruzzi, Italy) for *E. coli* and Baird–Parker Agar Base (Liofilchem, Italy) for *S. aureus*, were prepared by dissolving each nutrient in autoclaved water before the sterilization process at 120 °C for 15 min. After the sterilization, to complete the Baird–Parker agar preparation procedure, this was cooled to 50 °C, and an emulsion of egg yolk (Liofilchem, Italy) containing potassium tellurite was added. Both culture media were poured directly into Petri dishes before their solidification. To activate the bacteria, pellets of the bacterial strains were dissolved in distilled saline water (0.9% NaCl) and diluted, obtaining bacterial suspensions of 10^5^ CFU/mL, which were plated on the respective solid agar media. Sterilized 200 mg disks of sample powder, previously ground and pressed, were positioned in the middle of the Petri plate and every dish was incubated. *E. coli* was incubated at 44 °C for 24 h while *S. aureus* was incubated at 36 °C for 48 h. After the incubation time, the IHDs were calculated. For each sample, to determine the mean and standard deviation, four measures of the diameter were taken.

### 2.7. Boiling Water Test

The boiling water test was performed according to [29] by putting the samples in boiling water for about 20 min. After this time, the samples were removed from the water and the possible cracks and/or breaks were evaluated. In this test, samples aged 56 days were used.

### 2.8. Mechanical Strength

The compressive strength was evaluated by using Zwick/Roell Z010 machinery with a test speed of 2.0 mm/min and a preload of 5 N. 30 cylindrical specimens for each formulation, with fixed dimension of 10 × 20 mm, were used. The tests were performed by placing the 28-day-aged specimens between two compressive plates, and a comparison with Portland cement was made according to the literature data. 

### 2.9. Moss Growing Test

**Figure 2:**
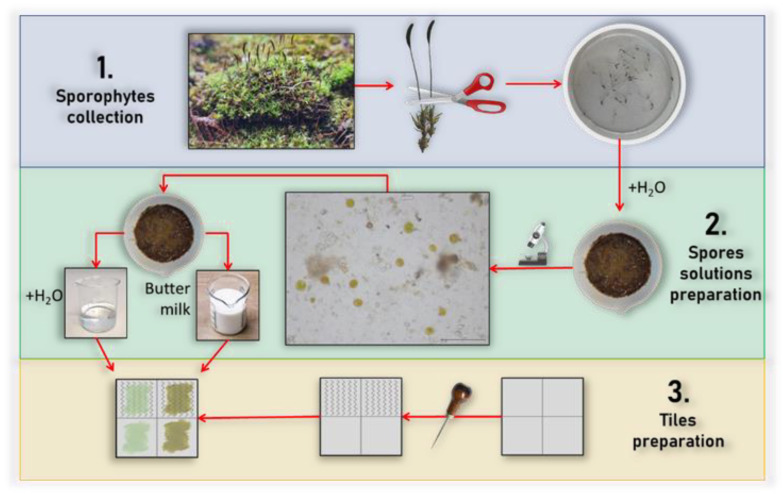
Flowchart showing the moss growing test procedure.

The moss growing test was performed to obtain information about the possibility of growing moss on geopolymers concrete. For this test, 15 × 15 cm sample tiles were used together with mosses collected from a concrete wall around the University of Campania “Luigi Vanvitelli”, Aversa (CE), Italy. The moss species were identified according to the literature [30] with a dichotomous key. The whole procedure of the test preparation can be divided into three steps:

1. Sporophytes collection. Once collected, the mosses were placed in a container and sprayed with H_2_O to increase the vitality of the spores. After 30 min of wetting them, the sporophytes containing the spores were removed using tweezers.
2. Spores’ solutions preparation. The collected sporophytes were put into a mortar containing 20 mL of water to help the pestling process and the sporophytes’ opening. The mixture was analyzed with an optical microscope Zeiss Standard 25, Germany, to confirm the presence of the spores. The amalgamated mixture was then divided into two halves: in one half another 10 mL of water was added, while in the second half, 10 mL of buttermilk was added. Buttermilk was made by whipping fresh cream for about 20 min and separating the solid part (the butter) from the liquid part (the buttermilk).
3. Tiles preparation. The tiles were realized and divided into four sections: two were left unaltered, while the other two were scratched to make the surface rough and simulate aging. The two mixtures realized in the previous step were brushed, as shown in the flowchart in Figure 2.

Once prepared, the tiles were left at room temperature and sprayed with water every day for three months to stay humid and stimulate the germination of spores.

## 3. Results and Discussion

### 3.1. Raw Materials Characterization

**Figure 3:**
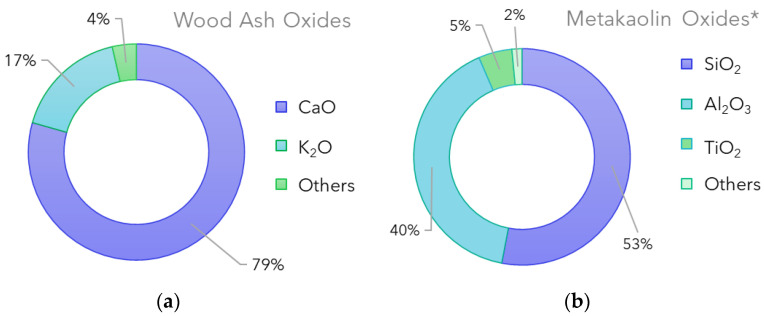
Metal oxides percentage present in (**a**) wood ash and (**b**) metakaolin. * Average values were adapted from [27].

XRF spectrometry results show the metal composition in oxides of the precursors used in this work. Metakaolin was previously characterized in [27]. Figure 3 reports the metal oxides present in both the metakaolin and the wood ash used.

Silica, alumina and titania were the main constituents of the metakaolin, while the wood ash was mainly composed of calcium and potassium. The metal composition of the raw materials is an essential parameter to consider when synthesizing geopolymers. The presence of silicon and aluminum in the metakaolin guarantees the formation of the oligomeric units, consisting of SiO_4_ and AlO_4_ tetrahedral geometry, in chains or rings that link together by sharing an oxygen atom. The tetrahedral geometry of the individual units allows the formation of the three-dimensional network, which is stabilized by metal cations (most commonly sodium, potassium, lithium or calcium) as well as bound water [31]. Because the ashes were added later in the synthesis, their involvement should be only of a filler nature; however, this cannot rule out their contribution, along with the sodium made available by the sodium hydroxide and sodium silicate solution, to the stabilization of the charges.

Given the nature of the ashes, the presence of inorganic and organic carbon was also evaluated. The results obtained highlight the absence of organic carbon and the presence of 9.35% of inorganic carbon. This result confirmed that the temperatures reached by the wood-fired ovens used for pizza making are high enough to burn all the organic matter.

### 3.2. Sample Characterization

The de-molding of the samples was performed after 7, 14, 28 and 56 days of aging. GP samples and GP10%WA specimens did not show significant differences after being removed from the molds at different aging times: the specimens were always humid and hard. No shrinkage phenomenon occurred, and the color was ivory and light grey for GP and GP10%WA, respectively. The GP20%WA sample exhibited the same hardness as GP and GP10%WA; however, the color was dark grey, and the de-molding process revealed its dryness. The GP30%WA sample de-molding also revealed a very dried dark grey specimen with no development of the shrinkage phenomenon.

**Figure 4:**
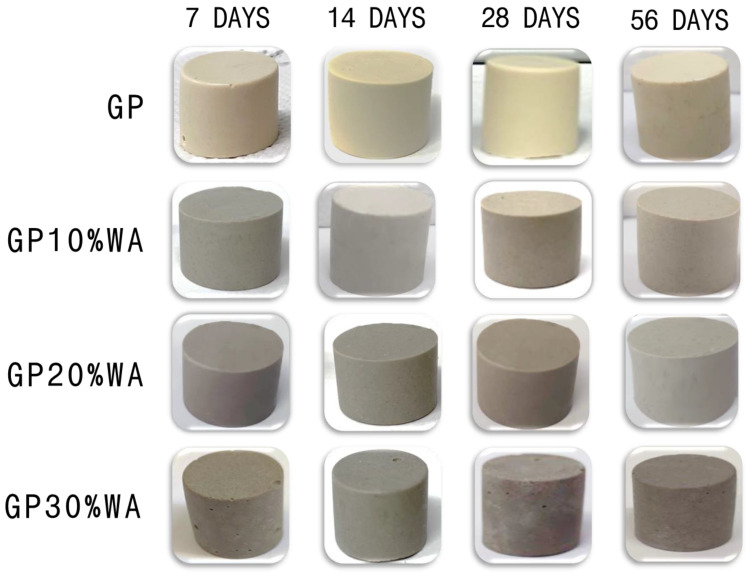
De-molded samples at different aging times.

Nevertheless, GP30%WA was the only sample that showed some physical changes during the time. As is pointed out in Figure 4, GP30%WA at 14, 28 and 56 days, compared to the one after seven days, formed a barely visible surface deposit caused by efflorescence. This phenomenon was primarily noticeable on the top surface of the specimen. Water evaporation on the exterior of geopolymer specimens induces moisture transportation, which moves the free alkalis from the matrix to the surface to react with the atmospheric CO_2_ [32]. According to Zhou et al. (2020), when a geopolymer cylinder’s curved surface is covered with plastic film, the efflorescence products are mainly formed on the top surface [33]. Another factor that influences the formation of efflorescence is the pore structure that correlates with moisture transportation. A high porosity increases moisture transportation, significantly enhancing free alkalis’ movement in the pore network [34]. Because the GP30%WA sample is the least malleable compared to the others (the higher amount of filler, the lower the water content), this led to the development of several air bubbles visibly present on the internal and external surface of the specimen, which partially justifies the phenomenon. Moreover, the formation of carbonates is justified by the large amount of Ca in the wood ashes, as confirmed by the XRF analysis and FT-IR analysis (Section 3.1 and Section 3.3). Thus, the efflorescence is mainly due to the formation of CaCO_3_ and Na_2_CO_3_ [35].

The density of samples was also evaluated after 56 days. The results obtained revealed how the increase in the wood ash content led to a decrease in sample densities. Indeed, the values obtained were 1.96, 1.85, 1.75 and 1.49 for GP, GP10%WA, GP20%WA and GP30%WA, respectively. 

Other information about the physical and chemical stability of the samples was obtained by the results of the integrity test, weight loss test, pH and ionic conductivity (IC) measurements (reported in Supplementary Materials). As shown in Table S1, the specimens resisted for 24 h in the water without losing structural integrity. The water appearance was clear, without any sediments. The only variation was recorded in the pH value of the water in which GP, GP10%WA and GP20%WA were soaked. The pH had a slight increment for these samples followed by a slight decrement after the test at 56 days. The pH value of GP30%WA was stable during all the tests. Generally, the pH values for specimens containing filler were higher than those that did not.

The graph in Figure S1 shows the result of the weight loss of the samples at different timings. All the samples had a weight loss after seven days of 1.05%, 2.00%, 2.82% and 5.61% for GP, GP10%WA, GP20%WA and GP30%WA, respectively. The higher weight loss is attributable to samples containing filler: as the quantity of the filler has increased, the weight loss percentage has increased, too. Indeed, the specimen that lost more weight was GP30%WA which is also the one with the higher percentage of filler. However, after 56 days of aging, the result revealed a weight loss lower than 1%, suggesting indirect information about the 3D network matrix stability. 

The graphs in Figure S2 show the result of the pH and conductivity of the samples de-molded at different timings. According to [28], an incomplete reaction between aluminosilicate powder and NaOH/Na_2_SiO_3_ solution causes fluctuation in the water’s pH and IC values after sample immersion. Therefore, the similar trend for all the specimens analyzed highlights the well-formed geopolymers. Generally, the pH value’s stabilization is registered after four hours for GP and GP10%WA, 6 h for GP20%WA and 2 h for GP30%WA. Due to the nature of the precursors used, the pH was alkaline with a value of about 12 for all specimens.

Regarding the ionic conductivity values, this followed the typical trend for alkali-activated materials [28]: an increase in ionic conductivity with time, due to the release of ions into the water, especially during the first 60 min of the analysis. Furthermore, the conductivity of GP10%WA, GP20%WA and GP30%WA samples was significantly higher than GP. This effect was presumably due to both the precursor and the filler. As the amount of filler increased, so did the conductivity. Wood ashes, as other studies [36] and XRF analysis confirmed, are rich in CaO that in an aqueous solution can convert into Ca(OH)_2_ and dissociate in Ca^2+^ and 2OH^−^ [37]. The former contributes to increasing the conductivity, and the latter increases the pH values. However, the conductivity of the 56-day-aged samples after 12 h reached the plateau, even if slower than the pH. The plateau value of the samples aged 56 days was about 320, 470, 590 and 772 mS/m for GP, GP10%WA, GP20%WA and GP30%WA, respectively.

### 3.3. FT-IR Analysis

**Figure 5:**
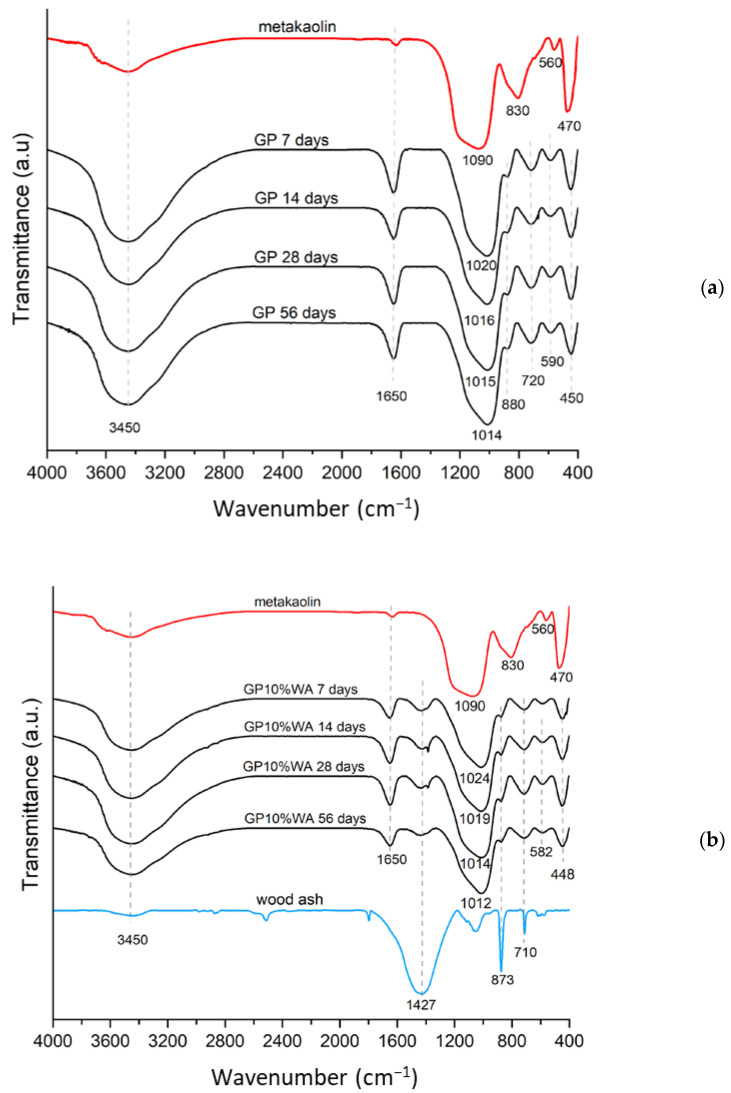

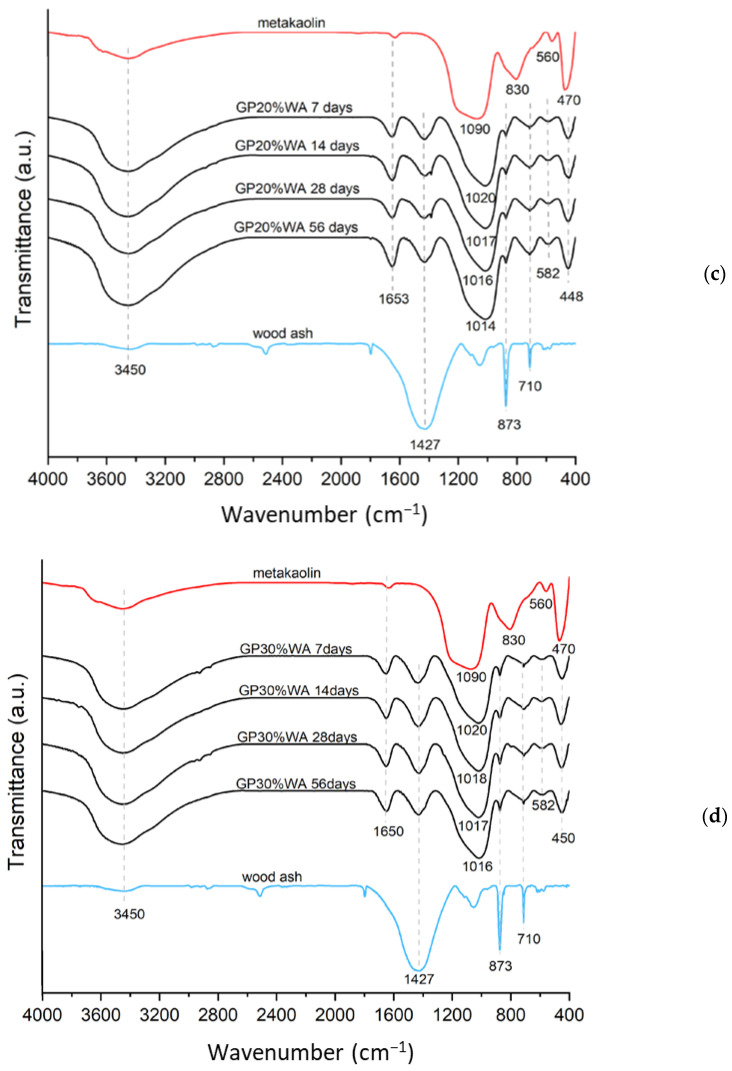
FT-IR spectra of samples during the time: (**a**) (GP); (**b**) (GP10%WA); (**c**) (GP20%WA); (**d**) (GP30%WA).

The FT-IR spectra of samples are shown in Figure 5 and Figure S3. The comparison of the FT-IR spectra of the geopolymers at different aging times with metakaolin allows the geopolymerization process to be tracked throughout time (Figure 5a–d). The characteristic band at 1090 cm^−1^ of metakaolin is assigned to the asymmetrical stretching of the Si-O-T bonds (where T = Si or Al) [38]. During geopolymerization, Si-O-Si bonds are replaced with Si-O-Al bonds, which have lower binding energy. Accordingly, the density state of peak maximum (DOSPM) shifts to lower wavenumbers in the samples (from 1090 cm^−1^ of MK to 1024–1012 cm^−1^ of the samples) and consequently confirms the occurrence of geopolymerization [39,40,41].

In all spectra, the bands at 1650 cm^−1^ and 3450 cm^−1^ are assigned to the presence of water in the geopolymer matrix. The former is related to the (H-O-H) bending while the latter is related to the hydroxyl (-OH) stretching. Water formation during the polycondensation reactions justifies the presence of a more significant amount of water in geopolymer samples compared to the metakaolin.

The spectra’s areas from 800 to 400 cm^−1^ also confirm the geopolymerization reaction: the bands are due to the vibrations of the Si-O and Al-O bonds. Minor variations in the position of the previous peaks in the various synthesized geopolymers are instead attributable to the change in the chemical environment following the polycondensation reactions. In particular, it is possible to observe small peaks at 873–880 cm^−1^, 710–720 cm^−1^, 582–590 cm^−1,^ and 448–450 cm^−1^. The first two are associated with the stretching vibration of the hexa-coordinate Al(VI)-OH and Al(VI)-O in metakaolin and with the bending vibration of tetra-coordinated Al(IV)-O-Si in a cyclic structure, respectively, while the last two are due to the Al-O-Si stretching vibrations and the Si-O-Si bending vibration, respectively [11,42].

Finally, another significant peak is located at 1427 cm^−1^ on samples synthesized with the wood ash filler and is probably due to the presence of the carbonate group in the wood ashes. According to the literature, this is precisely due to the stretching of C-O-C in the carbonate group [40,41,42].

### 3.4. Antibacterial Activity

**Figure 6:**
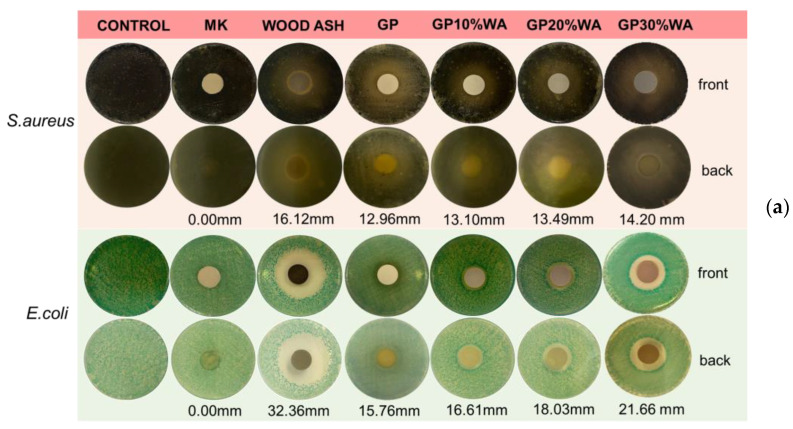

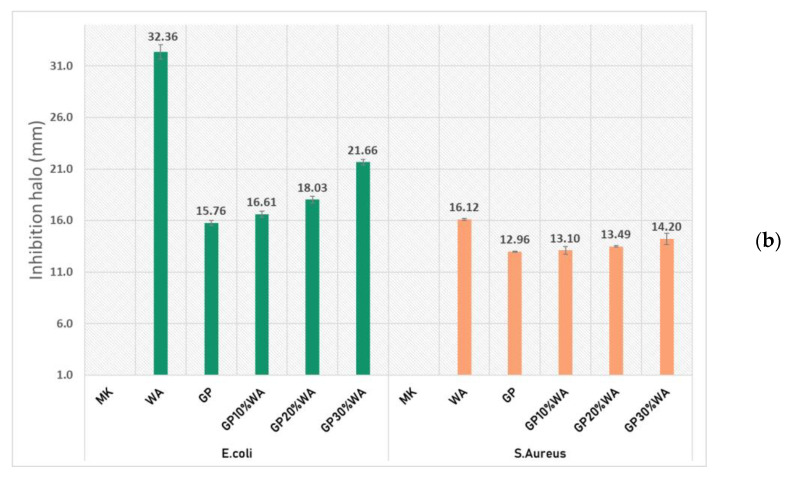
Antibacterial test results: (**a**) Petri plate results; (**b**) Graphical representation of inhibition halos’ measurements.

The material’s ability to inhibit bacterial growth is a preferential requirement for some applications. Figure 6a,b show the result of the antibacterial test. The observation of the Petri plates revealed that the metakaolin powder alone does not have antibacterial activity, and there are no inhibition halos for *S. aureus* nor for *E. coli*. Contrarily, wood ash powder can inhibit both bacteria growths. By comparing the inhibition halos’ measurements, the power of inhibition against *E. coli* is double with respect to *S. aureus*; the halo against the gram-negative bacterium is 32.36 mm while it is 16.12 mm against the gram-positive bacterium.

Regarding the samples, all of them showed antibacterial activity. In particular, specimens containing wood ash filler showed a higher and dose-dependent inhibition power. This behavior could be attributed to several reasons. First, most bacteria can live and multiply within pH 5–8, while the bacteria growing at pH above 10 are very few [43]. Therefore, with this explanation, it is reasonable to think that the geopolymer can create hostile bacterial growth environments.

Furthermore, the wood ash maintains a high pH value, increasing the inhibition power of the specimens. Another reason that could lead to the higher inhibition power of the samples containing wood ash is related to their large amount of calcium content [44]. Different authors reported the antibacterial effect of CaO on bacteria such *E. coli* and *S. aureus* [45,46,47]. As stated, the reaction between CaO and water generates Ca(OH)_2_ that dissociates into Ca^2+^ and 2OH^-^. Liang et al. reported that Ca^2+^ is responsible for disrupting the charge balance of the bacterial cell membrane and subsequently causes bacterial death. At the same time, OH^−^, in addition to increasing pH, may capture electrons on bacterial membranes obstructing the respiratory electron transport chain; hence, ATP production via energy metabolism is difficult, and bacteria lose activity [48]. Another reason for the antibacterial activity of wood ash could be related to the concept of bacterial adhesion. According to [49], bacterial adhesion is the first step in biofilm formation, and it is a survival mechanism that allows the collection of nutrients on surfaces. Therefore, high amounts of calcium may stimulate or reduce biofilm formation in different bacterial species. This condition was found to regulate biofilm development in *S. aureus* bacteria negatively. In particular, *S. aureus* produces several surface adhesins, such as clumping factors A and B (ClfA and ClfB) and biofilm-associated protein (Bap). These proteins contain Ca^2+^-binding EF-hand-like motifs, and binding the ion inhibits their role in cell adhesion and biofilm formation.

### 3.5. Boiling Water Test

**Figure 7:**
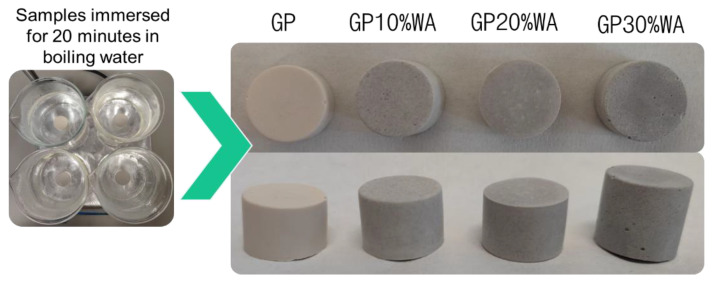
Image showing the result of the boiling water test.

Boiling water is one of the most severe tests, requesting only one run cycle. According to [29], fully geopolymeric materials resist in boiling water and remain intact. Therefore, this test was performed to validate the result obtained with FT-IR regarding the occurrence of the geopolymerization process. From the results (Figure 7), it emerged that all the specimens analyzed have passed the test with no cracks/breaks on the surface. All the specimens remain unbroken, confirming the solid internal structure of specimens and the complete occurrence of the geopolymerization process. The water was also checked, and it always appeared clear, with no suspensions inside.

### 3.6. Mechanical Strength

**Figure 8:**
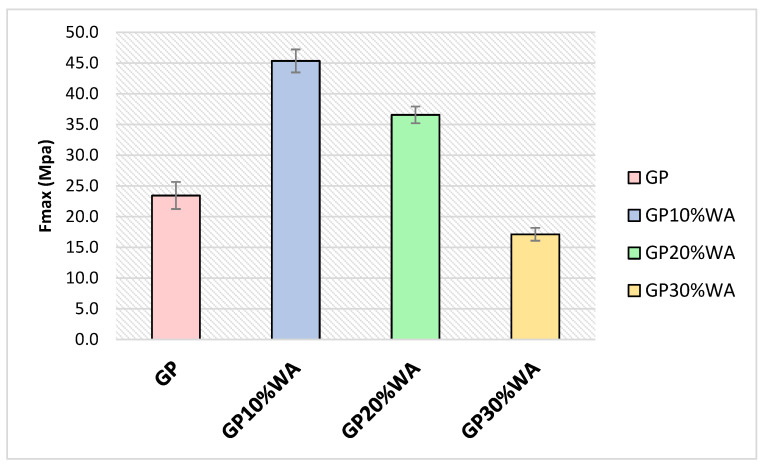
Mechanical compressive strength results.

Figure 8 shows the results of the compression tests of the samples GP, GP10%WA, GP20%WA and GP30%Q performed after 28 days of synthesis. All the samples showed good mechanical properties, comparable to the results in the literature of the compression tests of ordinary Portland cement [50,51,52]. Furthermore, a study by M. Abdullahi [53] reported the compressive strength of wood ash/OPC concrete specimens. The author realized various formulations by substituting a certain amount of OPC with wood ash (from 0 to 40 wt%) and evaluated the mechanical properties after 28 and 60 days. From the result, adding 10, 20, 30 and 40% of wood ash always decreased the mechanical strength compared to the OPC specimen without addition. Contrarily, from our results, samples containing the wood ash filler were more resistant compared to the filler-free specimens. In particular, the sample that showed the greatest mechanical strength was GP10%WA with an average compressive strength of 45.34 MPa. A similar result was achieved by GP20%WA with an average compressive force of 36.55 MPa. In contrast, GP30%WA scored a lower mechanical strength, but this result could be justified by the fact that the dough of this formulation was less malleable and so more prone to bubble formation during the mold casting procedure. Due to the water demand of wood ashes, the mixtures of GP20%WA and in particular GP30%WA were more difficult to work with, leading to the formation of a less fluid and more viscous paste [36]. For this reason, as the filler increased, the molding was also not optimal, and small bubbles were visibly present on the surface of GP30%WA. In conclusion, it can be said that wood ashes, as confirmed by the XRF analysis, being rich in calcium, lead to the formation of a more resistant geopolymer, both in the case of GP10%WA and GP20%WA.

### 3.7. Moss Growing Tests

**Figure 9:**
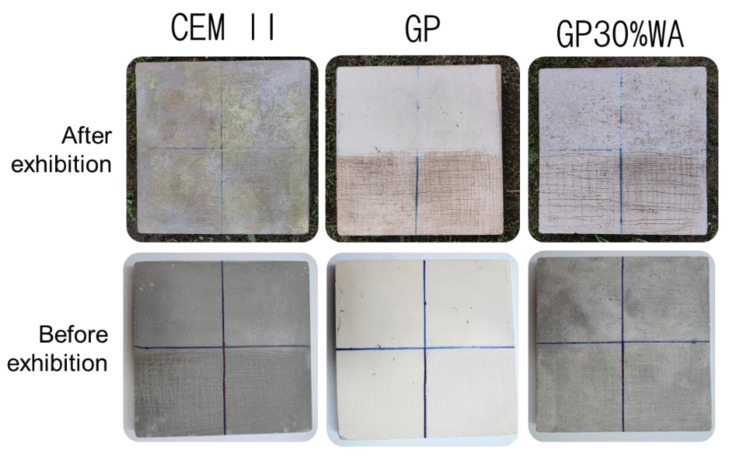
Moss growing test results before and after the exhibition.

Figure 9 shows the results of the moss growing test. Unlike the concrete tile (CEM II) in which moss growth occurred, none of the geopolymeric tiles with (GP30%WA) and without filler (GP) did this growth. Geopolymeric material and wood ash, having a basic pH, may create a hostile environment for the formation of the bacterial layer [43]. It is known that the bacterial layer is responsible for the engraftment of the spores [54]. Therefore, this could explain why the moss growth did not occur on geopolymers with and without filler [55]. In this experiment, an attempt was made to help the formation of the bacterial layer, using buttermilk and scratches on the surface, but these two parameters would not seem to have affected the final result either regarding the geopolymers or regarding the cement. The formation of the bacterial layer and, therefore, the moss’s engraftment on the cement tile, took place in a fairly homogeneous way, regardless of the scratches and the area treated with the buttermilk. These results may suggest that geopolymeric material can last longer in the environment without losing its properties and without being affected by plant organisms, therefore giving preliminary information about the durability of the synthesized materials.

## 4. Conclusions

Geopolymers are a promising technology for waste management, including wood ash. In this study, wood ashes from a local pizzeria were efficiently added as a filler for synthesizing geopolymers. All the results could be summarized as follows:

- The samples obtained with 10%, 20% and 30% of WA filler proved compact and solid, despite some surface bubbles in the GP30%WA formulation.
- The similar trend of pH and conductivity tests for all the samples highlighted the well-formed geopolymers. All species passed the integrity test, remaining stable to treatment in water for 24 h. This behavior was also confirmed by weight loss tests, whose percentage values do not exceed 1% after 56 days of curing.
- FT-IR analysis, as well as the boiling water test, confirmed the occurrence of the geopolymerization process in all the samples.
- The antibacterial analysis, as well as the moss growing test, demonstrated how geopolymers, with and without wood, can create a hostile environment for the formation of the bacterial layer and consequently the growth of mosses. This suggests their higher durability, compared to ordinary cement, over time, and their application in outdoor environments. The mechanical tests have shown how adding wood ash forms a stronger geopolymer than ash-free ones.

From the results obtained, the synthesized materials have been shown to possess part of the characteristics necessary for being applied in outdoor environments. Further studies are needed to evaluate the complete feasibility of their use.

## Figures and Tables

**Table 1:** Composition of the samples synthesized and W/S ratios.

Sample	Metakaolin	Wood Ash	W/S
GP	100%	0%	0.360
GP10%WA	90%	10%	0.358
GP20%WA	80%	20%	0.328
GP30%WA	70%	30%	0.309

## Data Availability

The data presented in this study are available on request from the corresponding author.

## Supplementary Materials

The following supporting information can be downloaded at: https://www.mdpi.com/article/10.3390/polym15071639/s1, Table S1: Results of the integrity test during the time; Figure S1: Result of the Weight Loss test during the time; Figure S2: Result of the Weight Loss test during the time; Figure S3: Result of the FT-IR analysis during the time.
