# Supplementary material for: Mosses on Geopolymers: Preliminary Durability Study and Chemical Characterization of Metakaolin-Based Geopolymers Filled with Wood Ash
Source: Polymers (Basel). 2023 Mar 25;15(7):1639. doi: 10.3390/polym15071639 (PMC10096776; doi:10.3390/polym15071639)
Supplement: Supplementary file 1 [file polymers-15-01639-s001.zip › polymers-2286585-supplementary.pdf]

Supplementary material

1. *Integrity test*

The integrity test was performed according to [1,2]. MilliQ water (1:100 solid-water ratio) was added to sample amounts, more or less 3 g. After 24 h, the water was removed, and the integrity was evaluated by estimating: i) the final pH solution; ii) hardness; iii) water transparency. The results are reported in Table S1.

Table S1. Results of the integrity test during the time.

Time (days)	7	14	28	56	
pH	8.2	9.2	9.55	8.75	**GP**
Resistance	hard	hard	hard	hard	
Water properties	clear	clear	clear	clear	
pH	8	9.88	10.05	9.84	**GP10%WA**
Resistance	hard	hard	hard	hard	
Water properties	clear	clear	clear	clear	
pH	7.8	10.4	10.21	9.98	**GP20%WA**
Resistance	hard	hard	hard	hard	
Water properties	clear	clear	clear	clear	
pH	10.34	10.09	10.28	10.10	**GP30%WA**
Resistance	hard	hard	hard	hard	
Water properties	clear	clear	clear	clear	

2. *Weight loss*

Sample amounts, more or less 4-5 g, were put in the beckers and completely covered with acetone (C_3H_6O). After 3h, the samples were removed from the acetone and left in the oven for another 3h. The oven was set up to 25°C, to mimic the room temperature. After 3h, the samples were weighted (W_i) and placed in beckers with MilliQ water as well as the integrity test condition. 24 h later, the water was removed, and the samples were again put in acetone for another 3h and then left again for 3h in the oven set to 25°C. At the end of this 3h, the weight of the sample was taken (W_f). The weight loss was calculated using equation (a) [2].

$$\text{Weight loss} = (W_i - W_f)/W_i \times 100 \qquad \text{(a)}$$

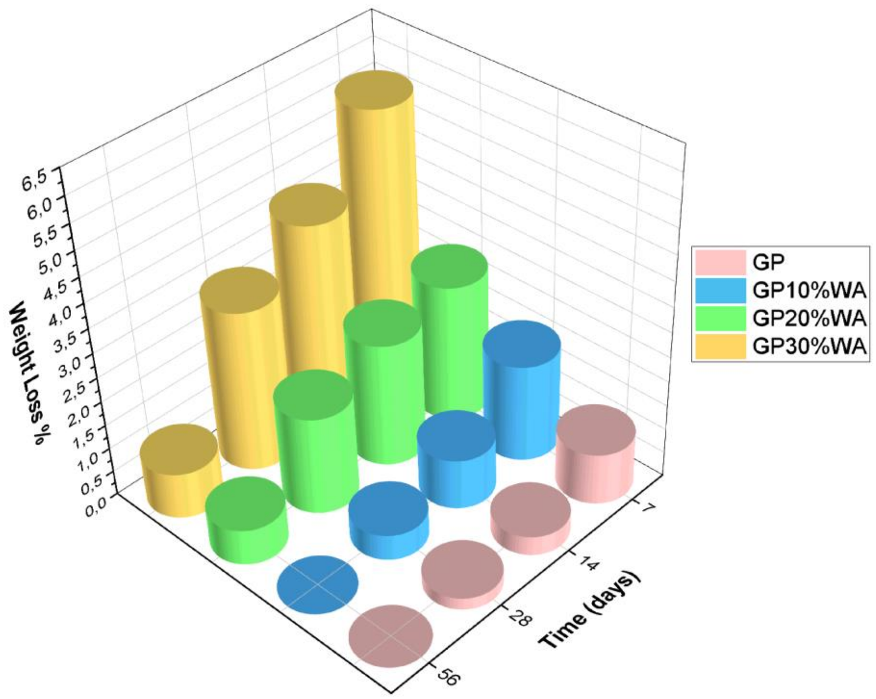

Figure S1. Result of the Weight Loss test during the time.

3. *Conductivity and pH measurements*

Conductivity and pH measurements were performed following the procedures in literature [1] after 7, 14, 28, and 56 days, with Crison GLP31 and Crison GLP 21, respectively. MilliQ water (1:10 solid-water ratio) was added to the samples previously broken into small coarse pieces and an average value was taken over the first 24h. The measurements over the 24hours were the following: t1 = 5min, t2 = 10 min, t3 = 30 min, t4 = 60 min, t5 = 2h, t6 = 4h, t7 = 6h, t8 = 8h, t9 = 10h, t10 = 12h and t11= 24h.

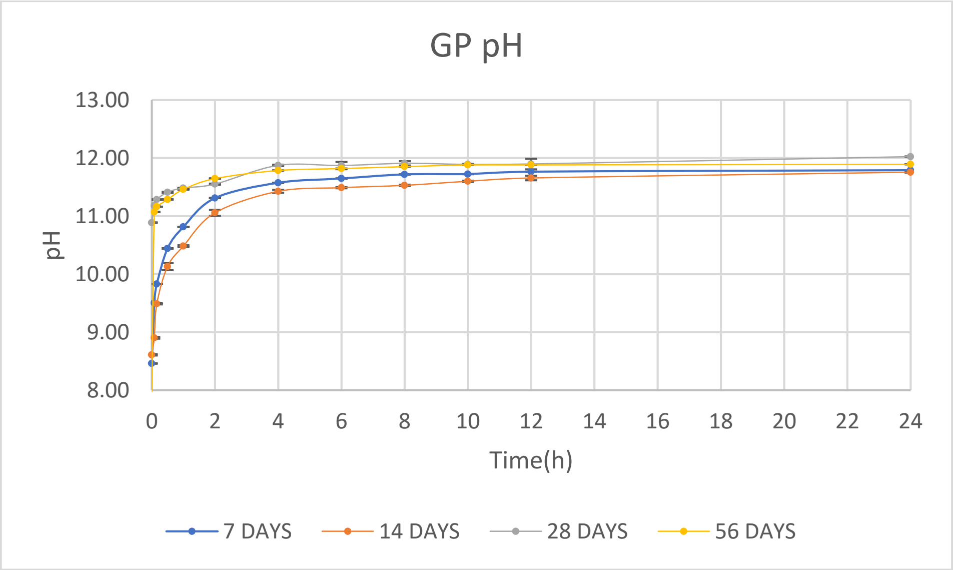

(a)

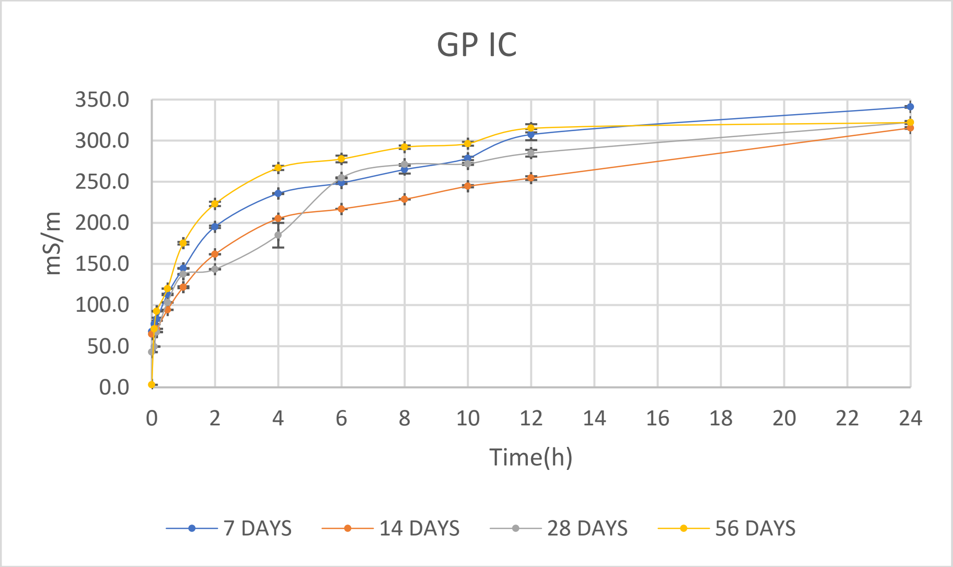
GP IC
mS/m
350.0
300.0
250.0
200.0
150.0
100.0
50.0
0.0
0 2 4 6 8 10 12 14 16 18 20 22 24
Time(h)
7 DAYS
14 DAYS
28 DAYS
56 DAYS

(b)

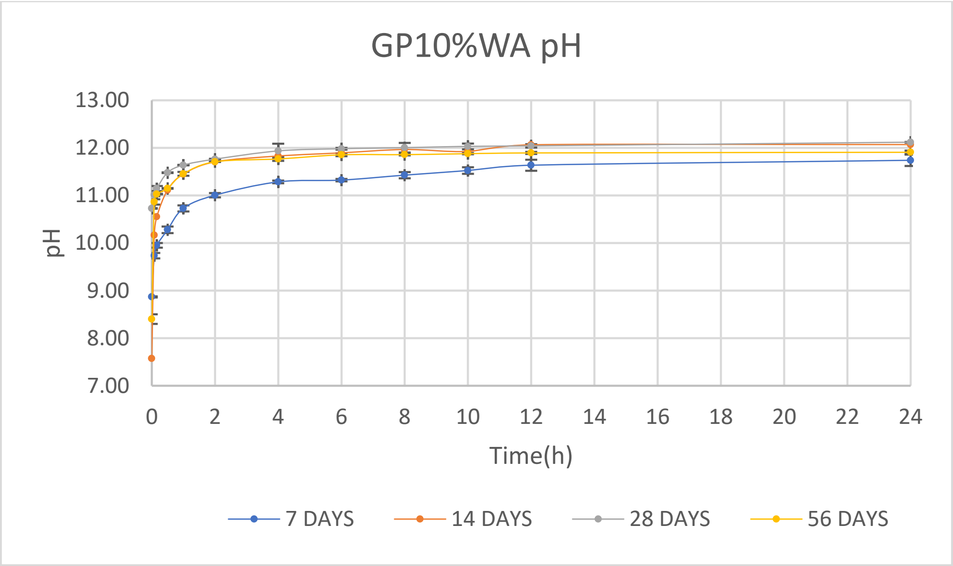
GP10%WA pH
pH
13.00
12.00
11.00
10.00
9.00
8.00
7.00
0 2 4 6 8 10 12 14 16 18 20 22 24
Time(h)
7 DAYS
14 DAYS
28 DAYS
56 DAYS

(c)

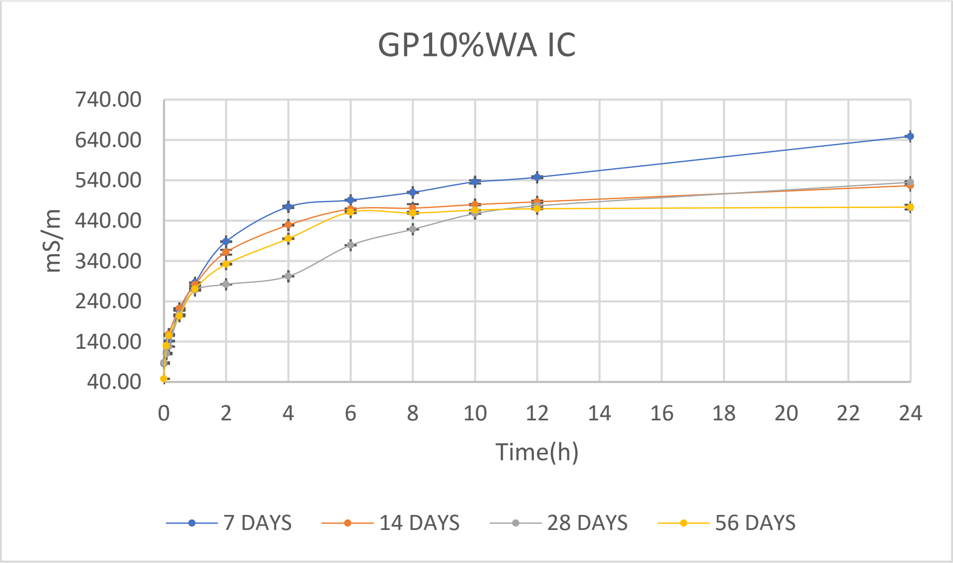
GP10%WA IC
mS/m
740.00
640.00
540.00
440.00
340.00
240.00
140.00
40.00
0 2 4 6 8 10 12 14 16 18 20 22 24
Time(h)
7 DAYS
14 DAYS
28 DAYS
56 DAYS

(d)

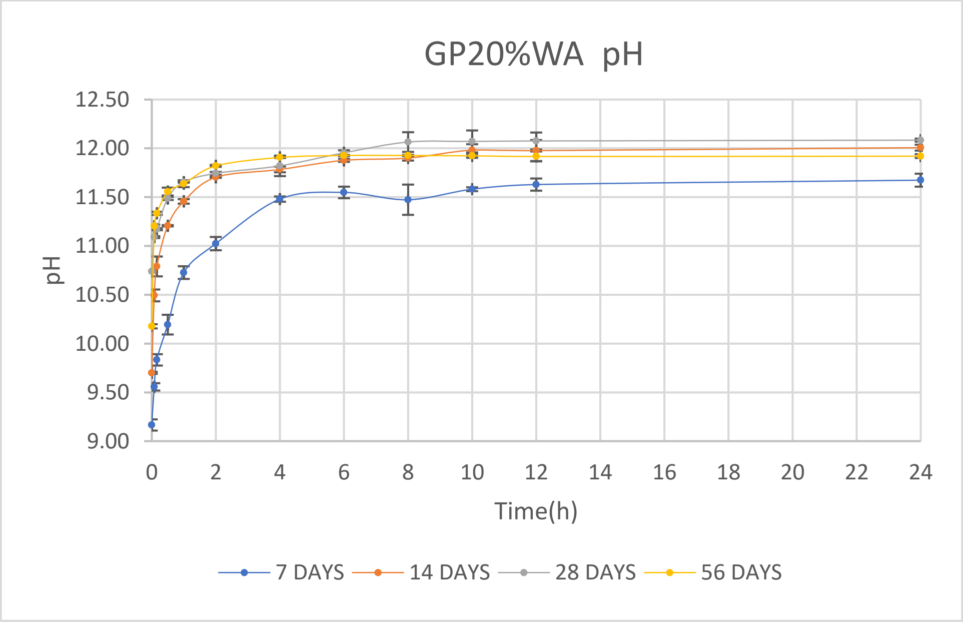
GP20%WA pH
pH
12.50
12.00
11.50
11.00
10.50
10.00
9.50
9.00
0 2 4 6 8 10 12 14 16 18 20 22 24
Time(h)
7 DAYS
14 DAYS
28 DAYS
56 DAYS

(e)

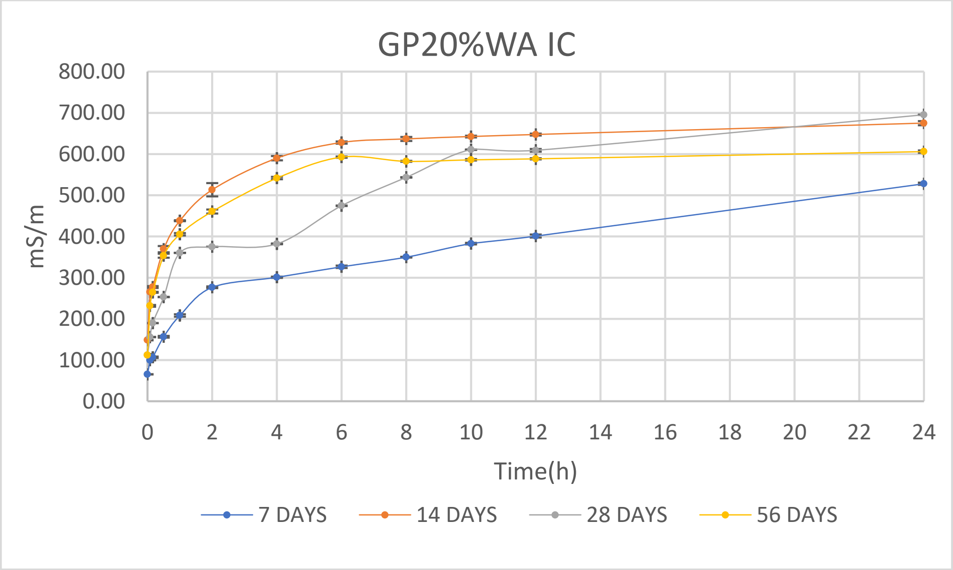
GP20%WA IC
mS/m
800.00
700.00
600.00
500.00
400.00
300.00
200.00
100.00
0.00
0 2 4 6 8 10 12 14 16 18 20 22 24
Time(h)
7 DAYS
14 DAYS
28 DAYS
56 DAYS

(f)

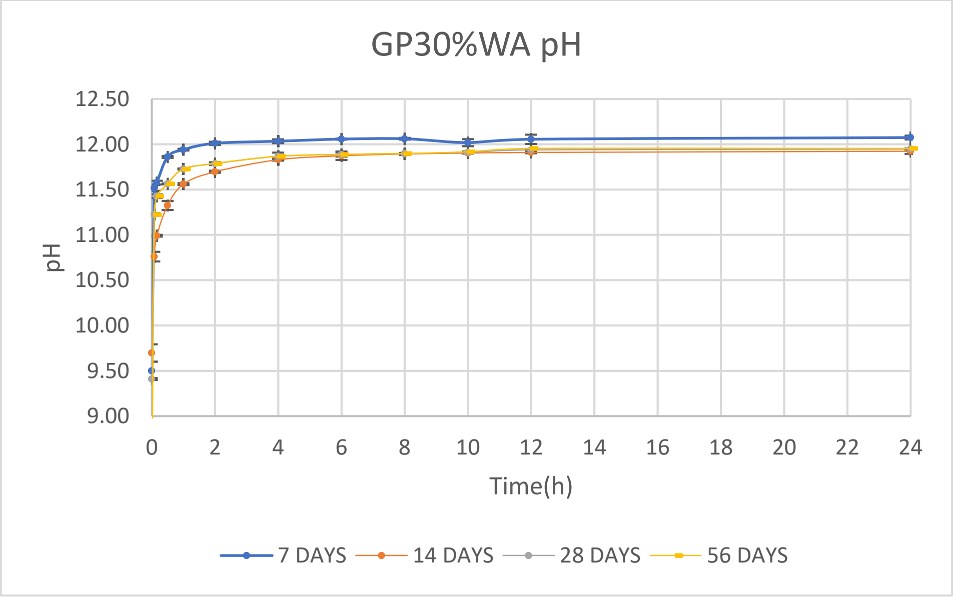
GP30%WA pH
pH
12.50
12.00
11.50
11.00
10.50
10.00
9.50
9.00
0 2 4 6 8 10 12 14 16 18 20 22 24
Time(h)
7 DAYS
14 DAYS
28 DAYS
56 DAYS

(g)

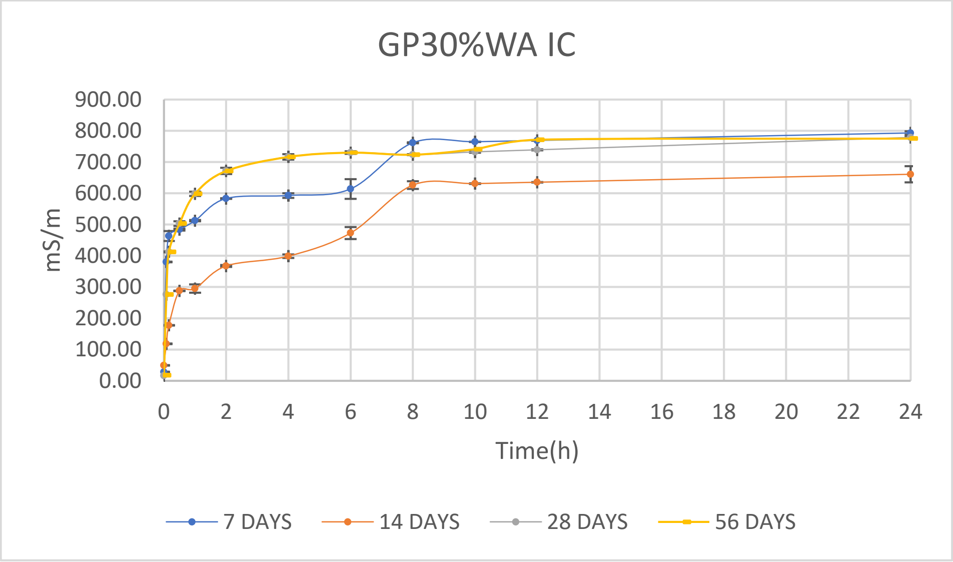

(h)

Figure S2. Result of the pH and Conductivity of GP (a,b); GP10%WA (c,d); GP20%WA (e,f); GP30%WA (g,h).

4. *FT-IR Analysis*

FT-IR analysis was performed with a Prestige21 Shimadzu machine equipped with a DTGS KBr (deuterated triglycine sulfate with potassium bromide windows) detector. The range of the performed analysis was between 400 and 4000 cm^{-1} and with a resolution of 2 cm^{-1} (60 scans). The KBr disks used for the analysis were realized with 2 mg of the sieved sample and 198 mg of KBr. FT-IR spectra were elaborated by IR-Solution and Origin software.

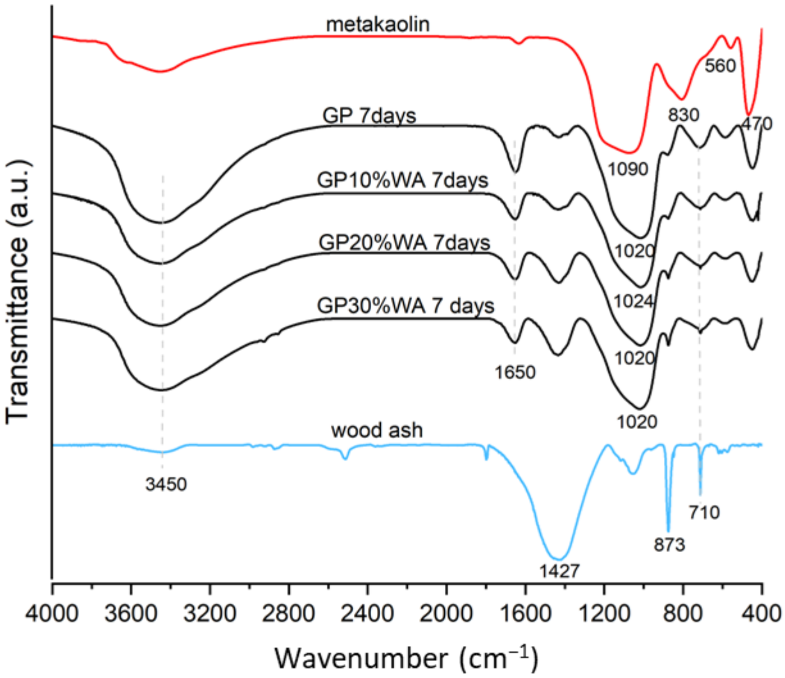

(a)

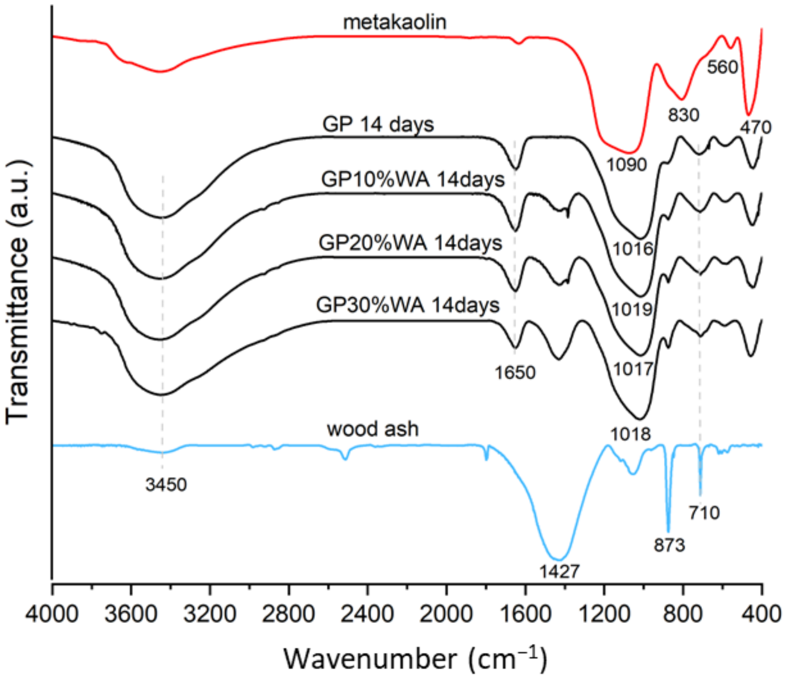
metakaolin
GP 14 days
GP10%WA 14days
GP20%WA 14days
GP30%WA 14days
wood ash
560
830
470
1090
1016
1019
1017
1018
1650
3450
1427
873
710
Transmittance (a.u.)
4000
3600
3200
2800
2400
2000
1600
1200
800
400
Wavenumber (cm⁻¹)

(b)

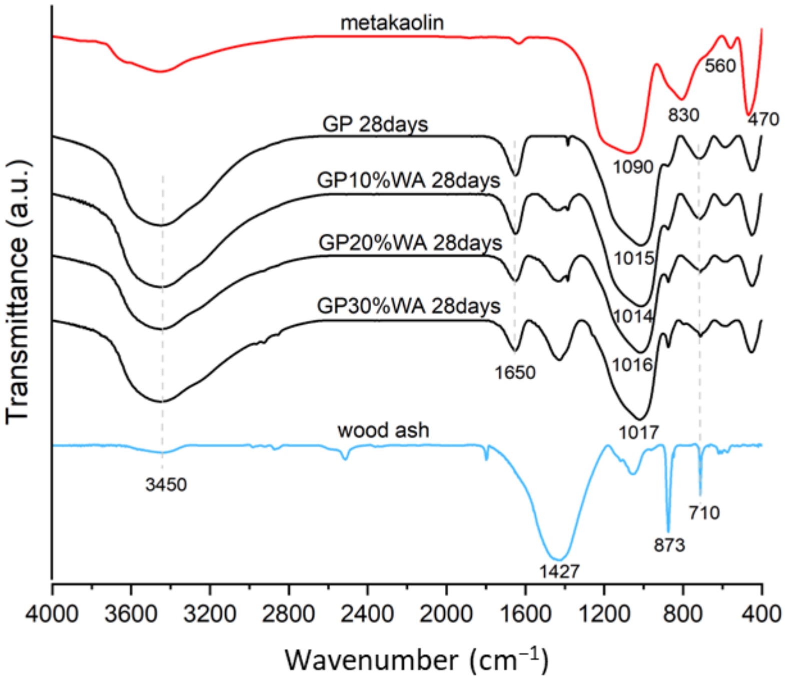
metakaolin
GP 28days
GP10%WA 28days
GP20%WA 28days
GP30%WA 28days
wood ash
560
830
470
1090
1015
1014
1016
1017
1650
3450
1427
873
710
Transmittance (a.u.)
4000
3600
3200
2800
2400
2000
1600
1200
800
400
Wavenumber (cm⁻¹)

(c)

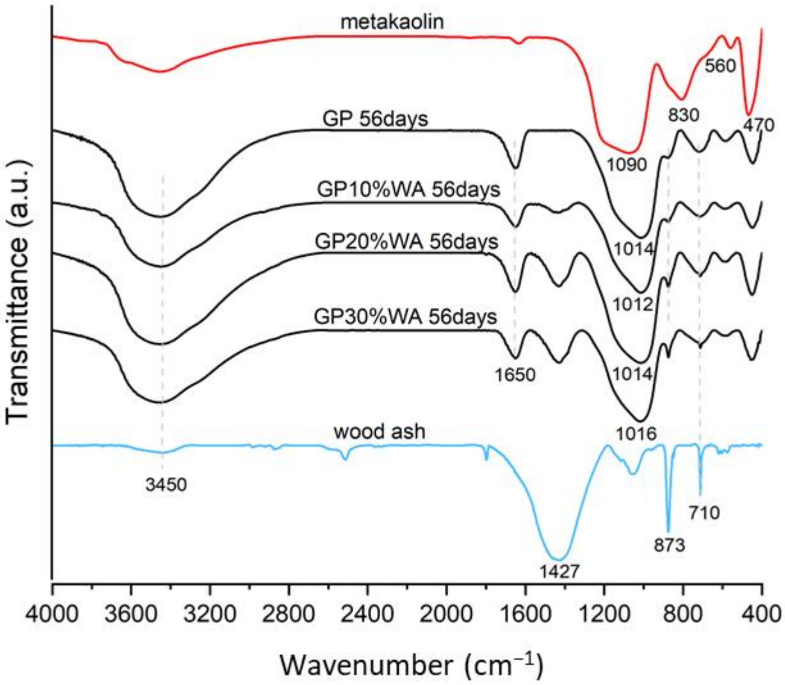

(d)

Figure S3. Comparison of FT-IR sample spectra at 7 days (a), 14 days (b), 28 days (c) and 56 days (d).

References

1. Sgarlata, C.; Formia, A.; Ferrari, F.; Leonelli, C. Effect of the Introduction of Reactive Fillers and Metakaolin in Waste Clay-Based Materials for Geopolymerization Processes. Molecules 2021, 26, 1325. https://doi.org/10.3390/molecules26051325.
2. D'Angelo, A.; Dal Poggetto, G.; Piccolella, S.; Leonelli, C.; Catauro, M. Characterisation of White Metakaolin-Based Geopolymers Doped with Synthetic Organic Dyes. Polymers 2022, 14, 3380. https://doi.org/10.3390/polym14163380.
